# Supplementary material for: Theoretical Study of Sphingomyelinases from Entamoeba histolytica and Trichomonas vaginalis Sheds Light on the Evolution of Enzymes Needed for Survival and Colonization
Source: Pathogens. 2025 Jan 5;14(1):32. doi: 10.3390/pathogens14010032 (PMC11768322; doi:10.3390/pathogens14010032)
Supplement: Supplementary file 1 [file pathogens-14-00032-s001.zip › Supplementary Figures 2024 final revision 2.pdf]

Theoretical study of sphingomyelinases from *Entamoeba histolytica* and *Trichomonas vaginalis* sheds light on the evolution of enzymes needed for survival and colonization.

Supplementary Figures

A

E. histolytica (B1N2L7–C4M9N5) · *T. vaginalis* (A2EEI1–XP_001579639.1) · *E. histolytica* (C4MA52)

	B1N2L7	B1N2O2	B1N4I9	C4LUW2	C4LVV3	C4LVW5	C4LYB8	C4LYZ5	C4M0T9	C4M5N7	C4M8T3	C4M962	C4M9N5	A2EEI1	A2FAR4	A2EE69	A2E5O5	XP_001296506.2	XP_001298660.1	XP_001296971.1	XP_001579639.1	C4MA52
tr|B1N2L7|Entamoeba_histolytica_(HM-1:IMSS)																						
tr|B1N2O2|Entamoeba_histolytica_(HM-1:IMSS)	0.03449																					
tr|B1N4I9|Entamoeba_histolytica_(HM-1:IMSS)	0.00660	0.04153																				
tr|C4LUW2|Entamoeba_histolytica_(HM-1:IMSS)	2.72130	2.64245	2.72130																			
tr|C4LVV3|Entamoeba_histolytica_(HM-1:IMSS)	2.94444	2.97893	2.94444	2.93511																		
tr|C4LVW5|Entamoeba_histolytica_(HM-1:IMSS)	2.77259	2.69124	2.77259	0.14792	3.06027																	
tr|C4LYB8|Entamoeba_histolytica_(HM-1:IMSS)	0.00329	0.03800	0.00329	2.72130	2.94444	2.77259																
tr|C4LYZ5|Entamoeba_histolytica_(HM-1:IMSS)	2.72130	2.69124	2.77259	2.82388	2.56975	2.82388	2.72130															
tr|C4M0T9|Entamoeba_histolytica_(HM-1:IMSS)	3.15208	3.38439	3.15208	3.12926	2.63906	3.06027	3.15208	2.67415														
tr|C4M5N7|Entamoeba_histolytica_(HM-1:IMSS)	2.67251	2.69124	2.67251	2.82388	3.04690	3.06027	2.67251	3.32743	2.87086													
tr|C4M8T3|Entamoeba_histolytica_(HM-1:IMSS)	2.58153	2.59593	2.58153	3.20337	2.92022	3.20337	2.58153	3.17805	2.74826	2.67906												
tr|C4M962|Entamoeba_histolytica_(HM-1:IMSS)	2.82666	2.79660	2.88381	2.87795	2.56735	2.93511	2.88381	2.20444	2.67415	2.77499	2.71543											
tr|C4M9N5|Entamoeba_histolytica_(HM-1:IMSS)	3.23212	3.28908	3.23212	2.67728	2.80468	2.82388	3.23212	2.94444	2.84913	3.04980	2.57288	2.64433										
tr|A2EEI1|Trichomonas_vaginalis_(G3)	3.25259	3.36153	3.11906	3.17805	3.36038	2.77259	3.11906	2.50286	2.71175	2.94092	2.61374	2.99573	2.66804									
tr|A2FAR4|Trichomonas_vaginalis_(G3)	2.36528	2.32008	2.30813	3.47093	2.75508	3.47093	2.30813	2.84781	2.58400	3.14631	2.81468	2.78356	2.77259	1.36498								
tr|A2EE69|Trichomonas_vaginalis_(G3)	2.42591	2.44524	2.36528	3.47093	2.81223	3.31678	2.36528	2.98134	2.63529	2.99215	2.69688	2.67233	2.99573	1.37711	0.42037							
tr|A2E5O5|Trichomonas_vaginalis_(G3)	2.59027	2.60639	2.59027	2.91777	2.74470	2.91777	2.59027	2.85438	2.82246	2.45674	2.82785	2.64696	2.51712	2.98631	2.54632	2.39476						
XP_001296506.2_Trichomonas_vaginalis_G3	2.77704	2.66723	2.77704	2.91985	3.10309	3.08690	2.77704	2.69892	3.35468	2.90872	3.19395	2.84246	2.89729	2.51022	2.18792	2.13073	1.34915					
XP_001298660.1_Trichomonas_vaginalis_G3	2.61496	2.63189	2.61496	2.82336	2.94193	2.88052	2.61496	2.87767	2.50355	2.75748	3.05243	2.97041	2.82477	2.74849	2.24374	2.24071	1.32176	0.95444				
XP_001296971.1_Trichomonas_vaginalis_G3	2.43900	2.48837	2.43900	2.97680	2.97893	3.11034	2.43900	2.67415	2.88531	3.01798	3.09104	2.56990	2.96636	2.38610	2.32654	2.35731	1.11748	1.16475	1.10085			
XP_001579639.1_Trichomonas_vaginalis_G3	2.66259	2.73231	2.66259	3.05085	2.53418	3.19395	2.66259	2.57973	2.89289	2.80215	2.89792	2.57728	2.42239	2.39201	2.06185	2.08538	1.13680	1.13114	1.16808	0.58226		
tr|C4MA52|Entamoeba_histolytica_(HM-1:IMSS)	2.82666	2.79660	2.88381	2.82388	1.65210	2.93511	2.88381	3.17560	2.50985	2.60762	3.01308	3.17560	2.72130	3.12090	2.87545	2.87545	2.73815	2.86537	3.10696	2.68305	2.58525	

B

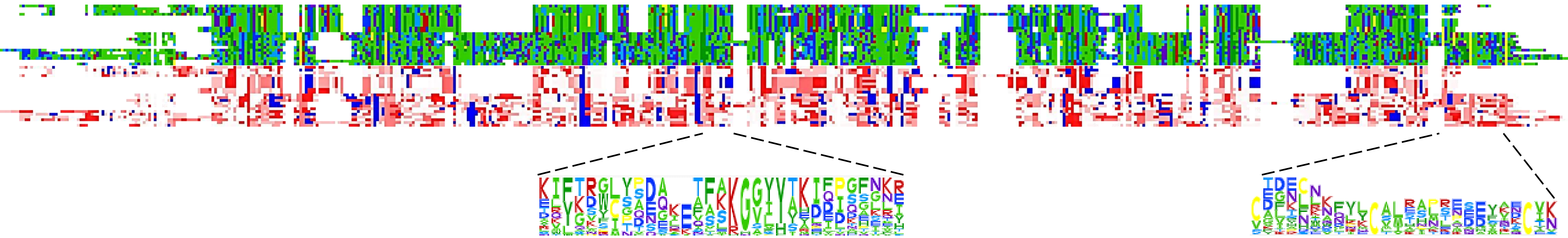

Supplementary Figure S1. SMases are enzymes with low sequence conservation. **Panel A** shows a pairwise com-parison between the amino acid sequence of SMase enzymes from *E. histolytica* and *T. vaginalis* annotated in UniProt or GenBank; the intensity of the color indicates enzymes with higher identity. **Panel B** shows pairwise alignments of all the enzymes from *E. histolytica* and *T. vaginalis*. The upper panel is in the MSA color scheme, and the lower panel is in the hydrophobicity color scheme. The two logos shown indicate only two regions with relatively conserved residues.

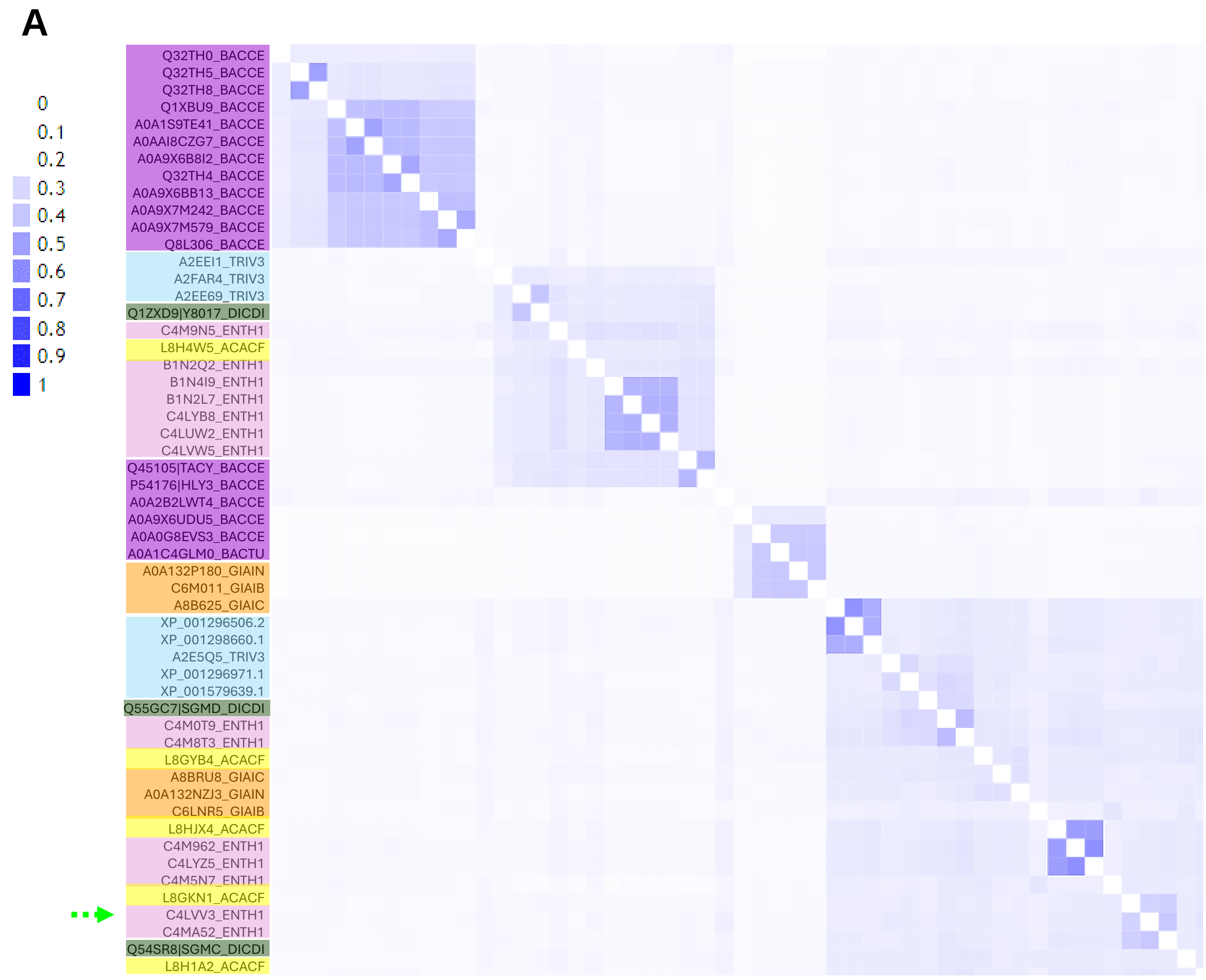

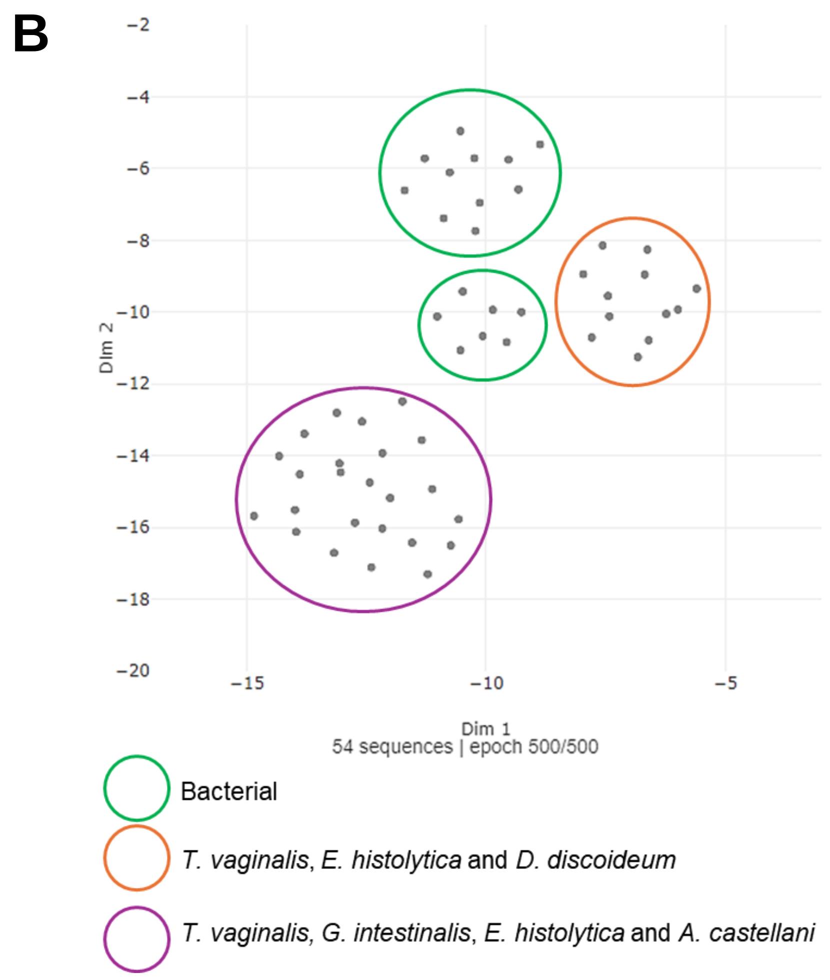

Supplementary Figure S2. Sequence comparison with examples of SMases from different organisms, including SMases with hemolysin activity, confirms low homology in these enzymes. **Panel A**, 2D pairwise identity map of representative examples of SMases. The color squares represent the organisms used. In pink, *E. histolytica*; in light blue, *T. vaginalis*; in orange, *Giardia* species (*intestinalis* and *lamblia*); in dark green, *Dictyostelium discoideum*; in yellow, *Acanthamoeba castellanii;* and purple, *Bacillus cereus* and *B. turingensis* SMases with hemolysin activity. **Panel B** shows a UMPA analysis of all sequences, as shown in **Panel A**. Color circles show the predominant grouping between organisms found in these sequences.

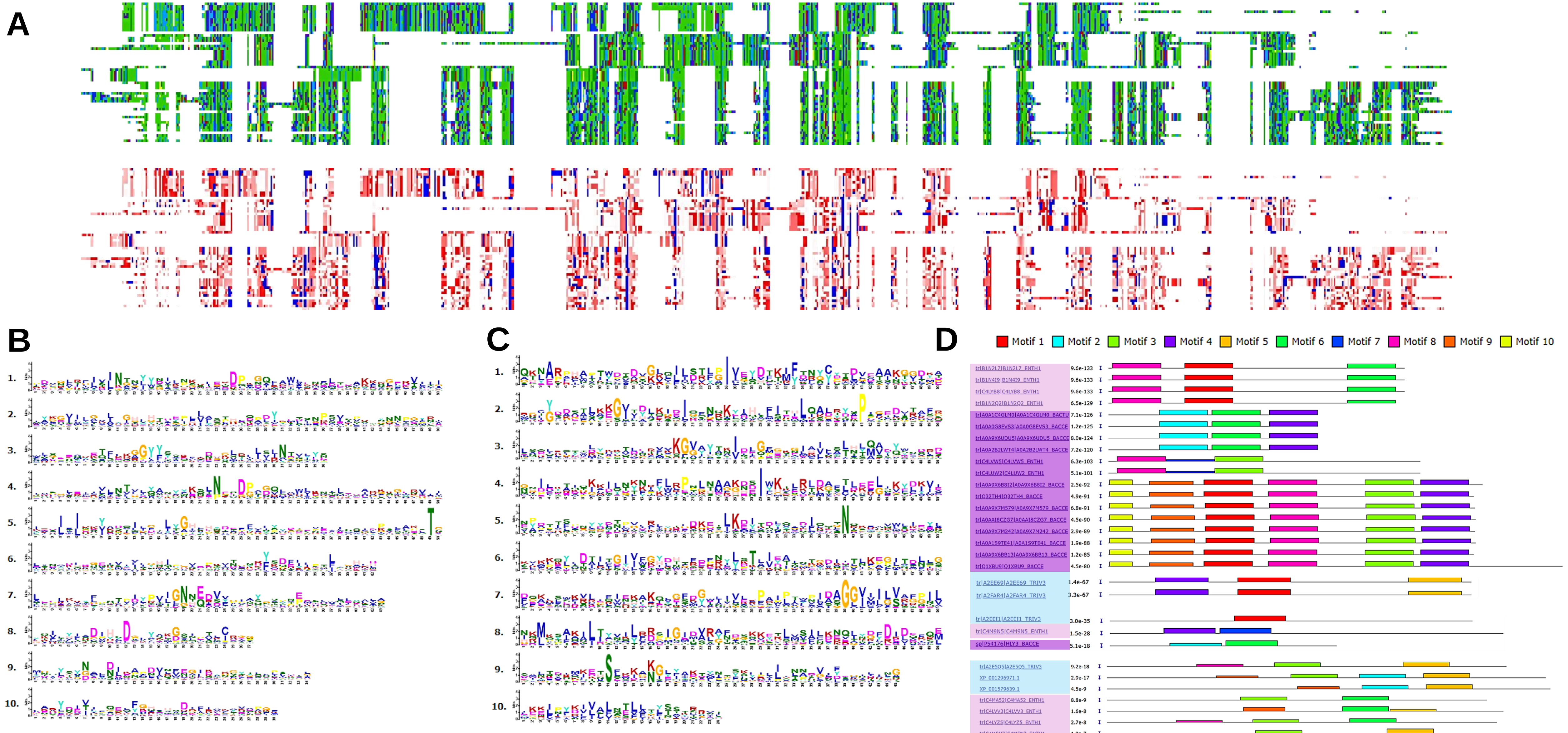

Supplementary Figure S3. No conserved motifs are found between SMase enzymes. **Panel A** shows the sequence comparison between all the sequences analyzed in **Supplementary Figure S2**, the upper panel in the MSA color scheme and the lower panel in the hydrophobicity color scheme. These sequences were analyzed for motif discovery using GLAM2, and **Panel B** shows the best ten motifs, ranging from 30 to 50 amino acids, with a score ranging from 0.12 to 0.33. **Panel C** shows the motif discovery results using GLAM2 analysis of only SMases from *E. histolytica*, *T. vaginalis*, and *B. cereus*, showing ten motifs of 24 to 50 amino acids with scores from 0.08 to 0.23. Motifs shown in **Panel C** were mapped against the protein sequence using the MAST tool, and the result is shown in **Panel D**, colors used: in pink, *E. histolytica*; in light blue, *T. vaginalis*, and in purple *B. cereus*.

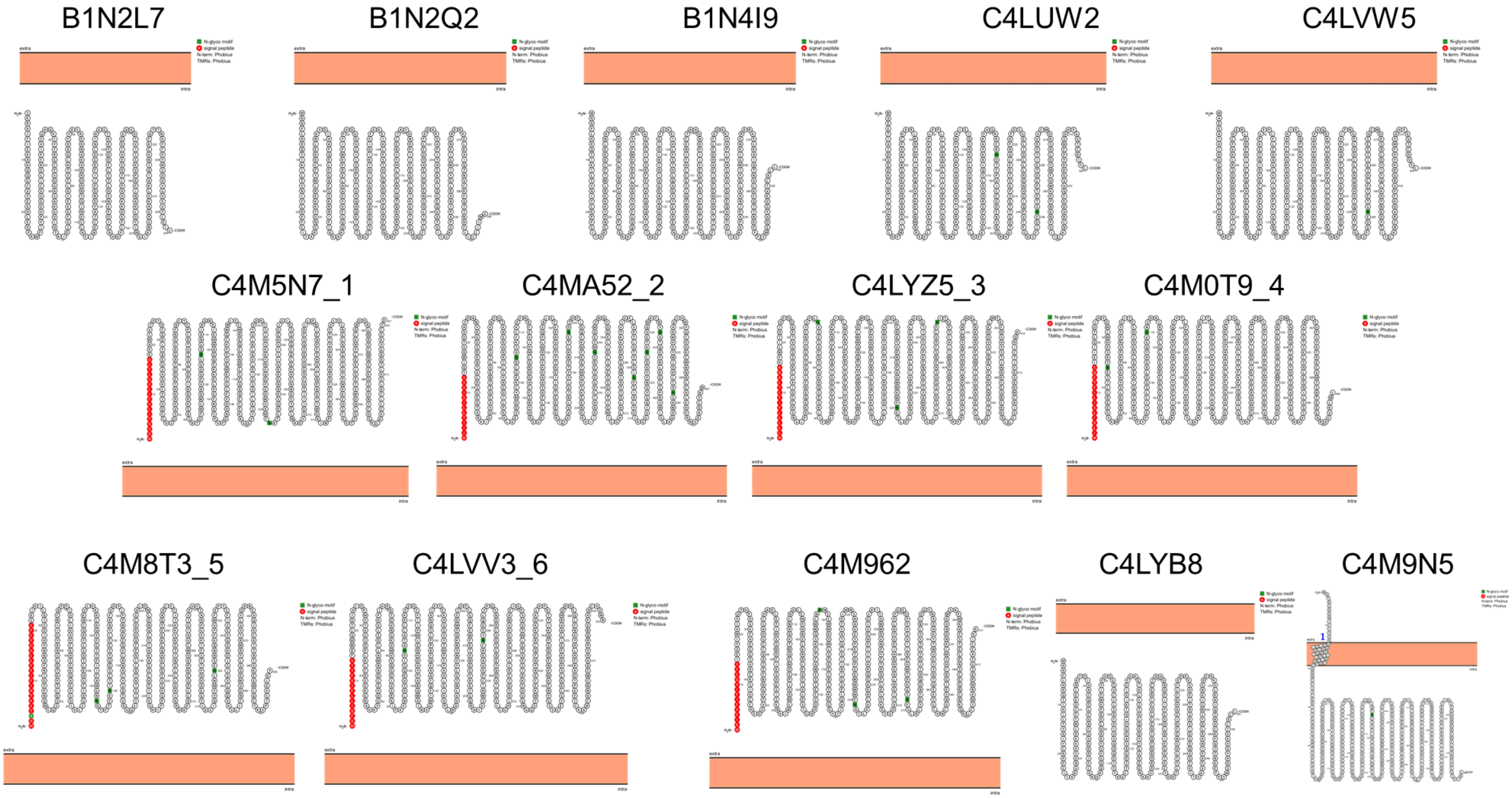

Supplementary Figure S4. Topology prediction of all annotated SMases from *E. histolytica* using Protter. The cell membrane is shown as a light orange bar. The predicted signal peptide is shown in red. Predicted glycosylation sites are indicated in green. UniProt accession numbers are shown.

Supplementary Table S1. Protein models quality parameters. For *T. vaginalis* only the models with Mg or oleic acid are shown.

Protein models	ipTM	pTM
SMase 6 no signal peptide	-	0.93
SMase 6 no signal peptide +Mg^{2+}	0.95	0.92
SMase 6 no signal peptide +Co^{2+}	0.92	0.91
SMase 6 no signal peptide, no c-terminal end, glycosylated	0.79	0.9
SMase 6 no signal peptide, no c-terminal end, glycosylated +Mg^{2+}	0.84	0.91
SMase 6 no signal peptide, no c-terminal end, glycosylated +Co^{2+}	0.79	0.9
SMase 6 no signal peptide, no c-terminal end, glycosylated +Zn^{2+}	0.77	0.88
SMase 6 no signal peptide, no c-terminal end, glycosylated +2xMg^{2+}	0.6	0.79
SMase 6 no signal peptide, no c-terminal end, glycosylated +2xCo^{2+}	0.58	0.77
SMase 6 no signal peptide, No C-terminal end, no loop, glycosylated	0.75	0.88
SMase 6 no signal peptide, No C-terminal end, no loop, glycosylated +2xMg^{2+}	0.8	0.9
SMase 6 no signal peptide, No C-terminal end, no loop, glycosylated +2xCo^{2+}	0.81	0.91
T. vaginalis A2EEI1 +2xMg^{2+}	0.9	092
T. vaginalis A2EEI1 +50 oleic acids	0.45	0.58
T. vaginalis A2FAR4 +2xMg^{2+}	0.91	0.94
T. vaginalis A2FAR4 +50 oleci acids	0.54	0.65
T. vaginalis A2EE69 +2xMg^{2+}	0.9	0.92
T. vaginalis A2EE69 +50 oleic acids	0.57	0.67
T. vaginalis A2E5Q5 +2xMg^{2+}	0.81	0.84
T. vaginalis A2EEI1 +oleic acids	0.25	0.44
T. vaginalis XP_001296506.2 +2xMg^{2+}	0.79	0.89
T. vaginalis XP_001298660.1 +2xMg^{2+}	097	0.83
T. vaginalis XP_001298660.1 +50 oleic acids	0.3	0.47
T. vaginalis XP_001296971.1 +2xMg^{2+}	0.97	0.82
T. vaginalis XP_001296971.1 +50 oleic acids	0.26	0.41
T. vaginalis XP_001579639.1 +2xMg^{2+}	0.96	0.81
T. vaginalis A2EEI1 XP_001579639.1 +50 oleic acids	0.21	0.4

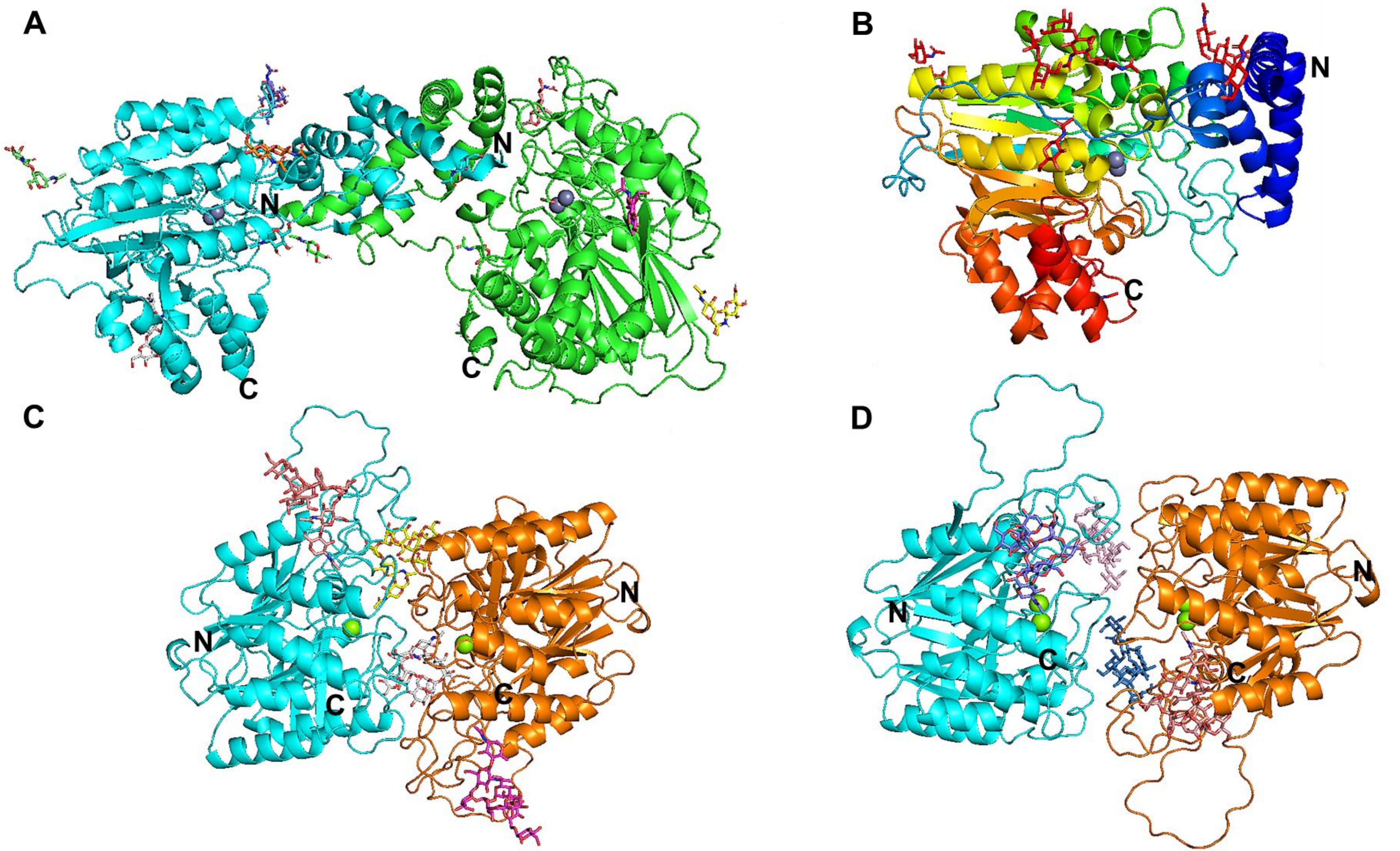

Supplementary Figure S5. Cartoon representation of reference human aSMase enzyme used. In **Panel A**, the dimer structure of human aSMase (PDB 5JG8), each subunit is shown in a different color. N and C terminal ends are indicated. **Panel B**, monomer of human aSMase enzyme (PDB 5I81) in rainbow color scheme. N and C-terminal ends are indicated. **Panels C** and **D** show the predicted *E. histolytica* aSMase6 model with one or two Mg+2 ions, respectively. Each monomer is shown in a different color, and the N and C-terminal ends are indicated.

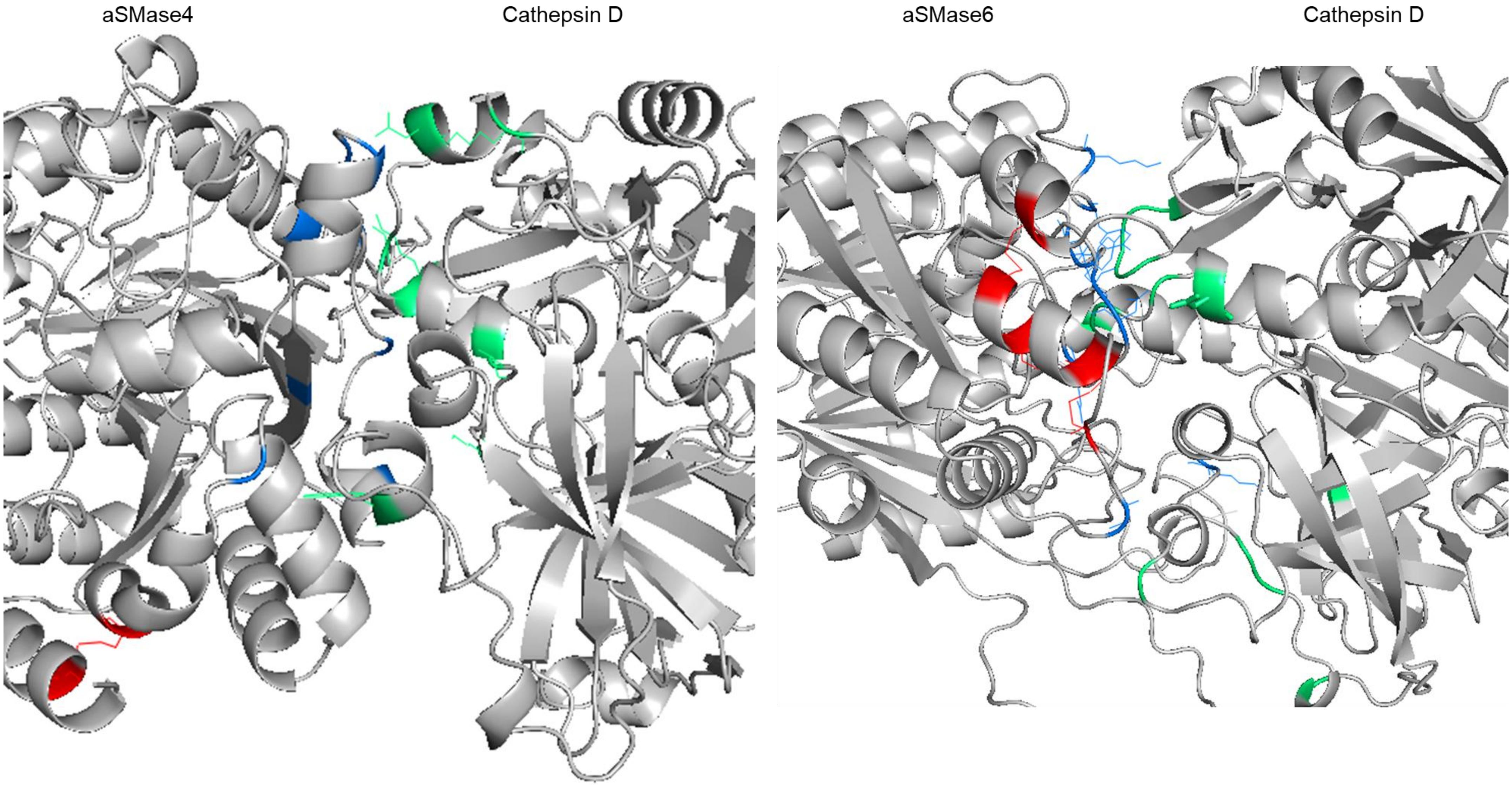

Supplementary Figure S6. AlphaFold3 models of aSMase4 and aSMase6 (as reference) with human cathepsin D. Each enzyme is indicated. In green, cathepsin residues are associated with the interaction and activity of the enzyme. In blue are the contact residues between the two proteins, in green the cathepsin D signature residues, and in red are the cysteines in the C-terminal end of the two aSMases.

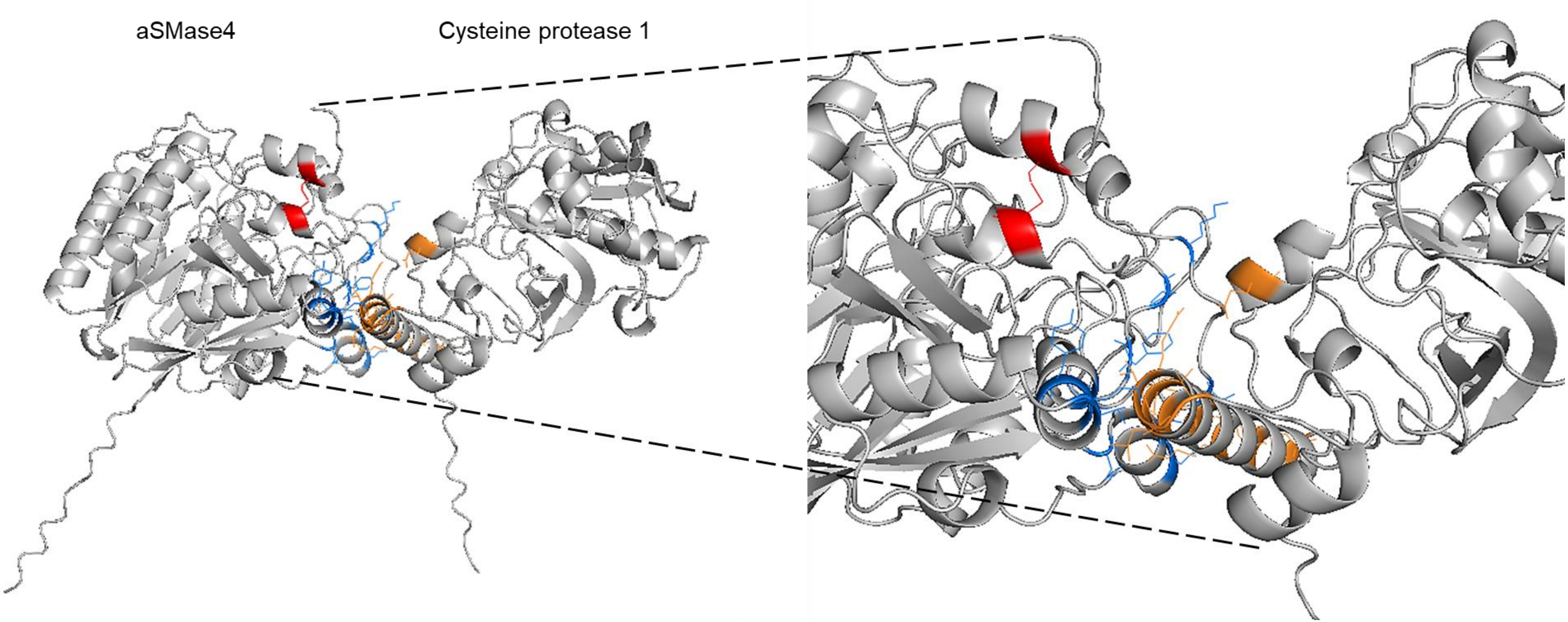

Supplementary Figure S7. AlphaFold3 models of aSMase4 and the putative processing enzyme cysteine protease 1 (UniProt Q01957). Contact residues from the aSMase4 enzyme are shown in blue, and from the cysteine protease 1 enzyme are in orange. aSMase4 cysteines are shown in red.

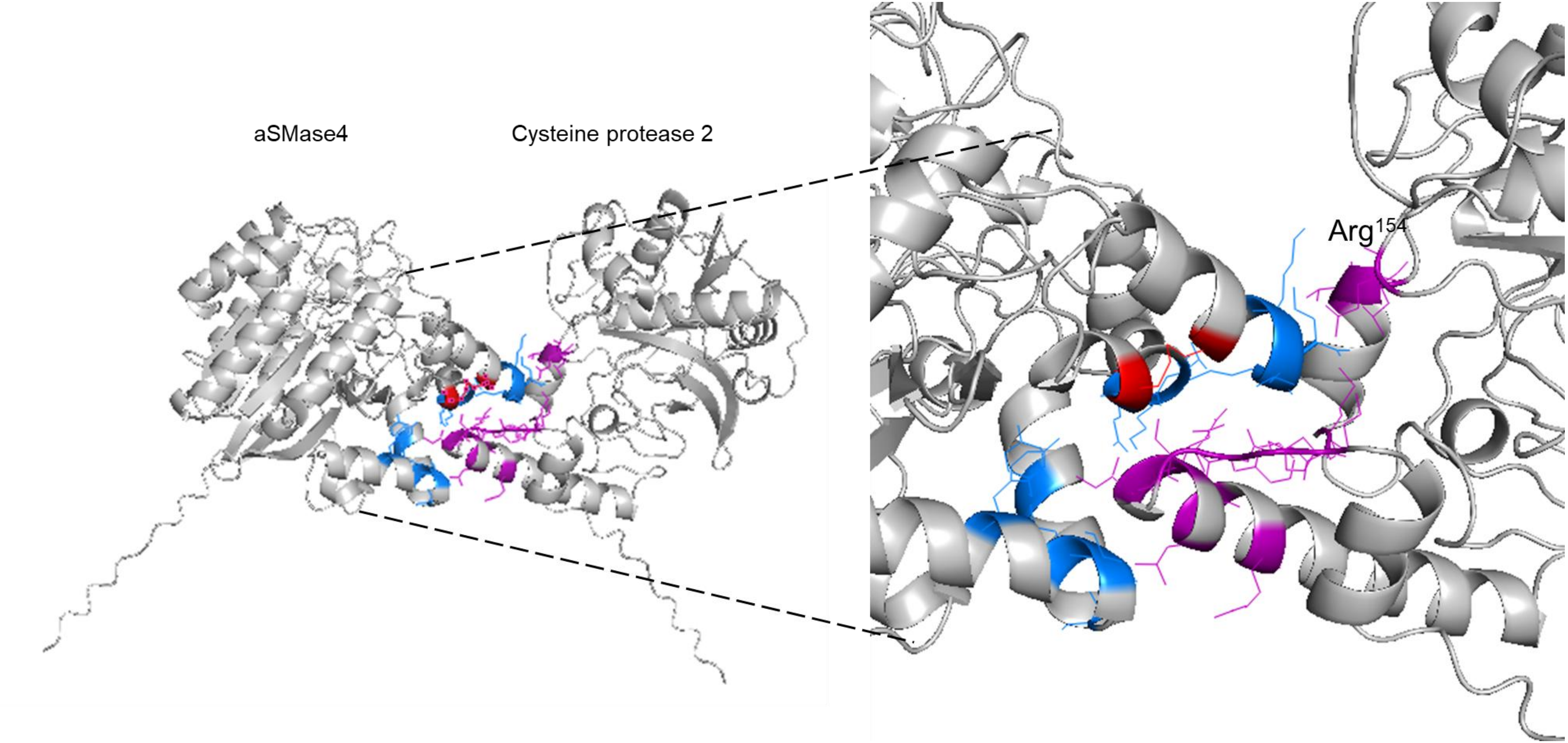

Supplementary Figure S8. AlphaFold3 models of aSMase4 and the putative processing enzyme cysteine protease 2 (UniProt Q01958). Contact residues from the aSMase4 enzyme are shown in blue, and the cysteine protease 2 enzyme is in purple. aSMase4 cysteines are shown in red.

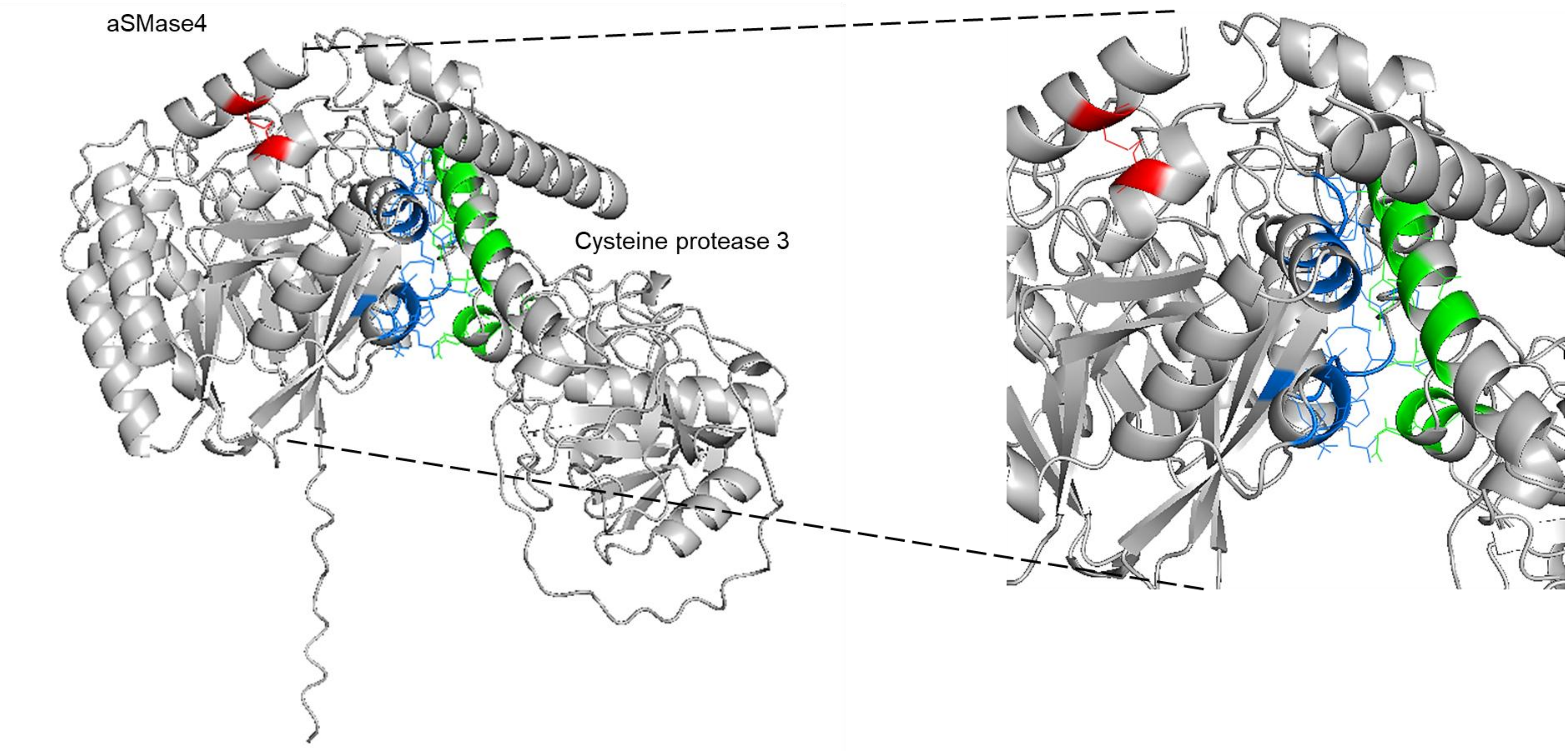

Supplementary Figure S9. AlphaFold3 models of aSMase4 and the putative processing enzyme cysteine protease 3 (UniProt P36184). Contact residues from the aSMase4 enzyme are shown in blue, and from the cysteine protease 3 enzyme are in green. aSMase4 cysteines are shown in red.

A

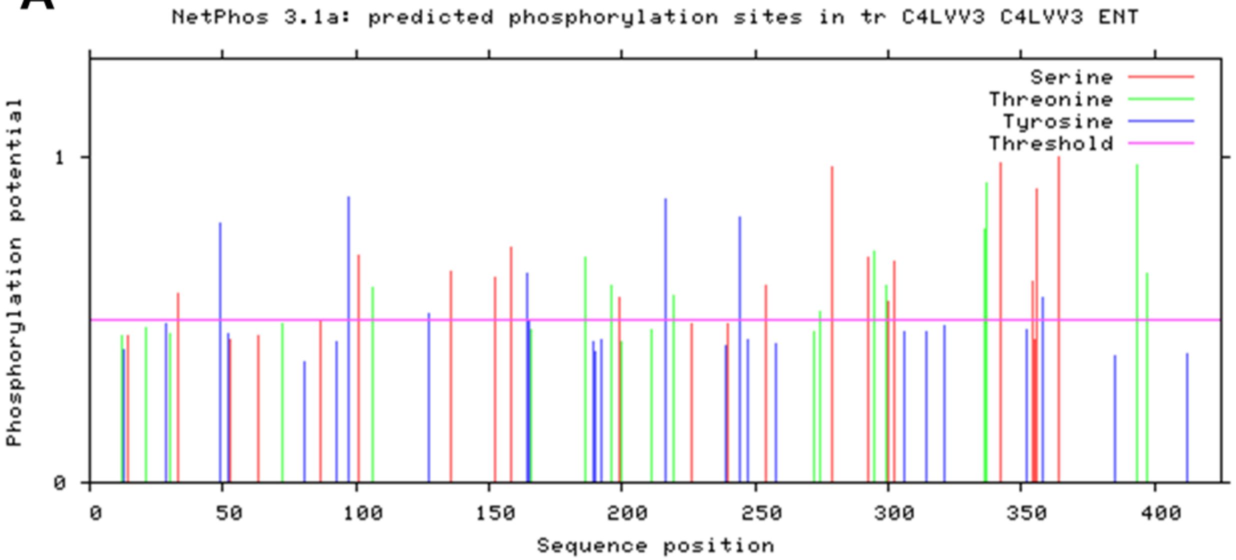

B

```
      MVFQILLFIVFTYSFEMWQLTDIHLDPMYTVGSDPLKLCREGKGNAGKYG    #    50
      DYSCDANMALLDSLVKFIKRNTDVNGKVMVYNGDIVSRAIPKYNLDYVKQ    #   100
      SIVNATALLKKFEDFFIIPMLGNHDVYPANQMAINSQWIFQFAAEQFAPF    #   150
      LSQNAIESFKHGGYYTMPFPAHLGIKVPLNAVVLNTVLYYNYNKQTMDST    #   200
      DPLGQFEWFKTVMDGYRKTGQRAIVSMHICPGVAERFNYSDQLYPQYNDR    #   250
      LVDSLAEYNDVILGVLCGHLHTDTFRILSKGNKKVMAFIGPSVDTWLGTS    #   300
      PSIRKYDVGNGQGYMKEFKNYHFQLRPKKDKLKPKTTIKQPSDGFNKWKF    #   350
      NYGSSSEYGLKELSPKEFEILAARMDKDLQLFNKYHLHFRADTPGFTCKG    #   400
      NCKNNCMCAVRYPRQNEFANCVVWN                             #   450
%1    ................................S...............Y.    #    50
%1    ..............................................Y...    #   100
%1    S....T....................Y........S..............    #   150
%1    .S.....S.....Y.....................T.........T..S.    #   200
%1    ...............Y..T........................Y......    #   250
%1    ...S...................T....S............S..T...TS    #   300
%1    .S.................................TT....S........    #   350
%1    ...S.S.Y.....S............................T...T...    #   400
%1    .........................
```

Supplementary Figure S10. NetPhos analysis of aSMase6 from *E. histolytica* revealed 33 putative phosphorylation sites. **Panel A** shows the position of the different phosphorylation sites. The horizontal pink line shows the threshold for the probability of each residue being phosphorylated. In **Panel B**, the aSMase6 sequence is shown, and the highest probability residues from **Panel A** are shown. The highest-probability residues were used for the models generated with AphaFold3, shown in **Figure 7 Panel C**.

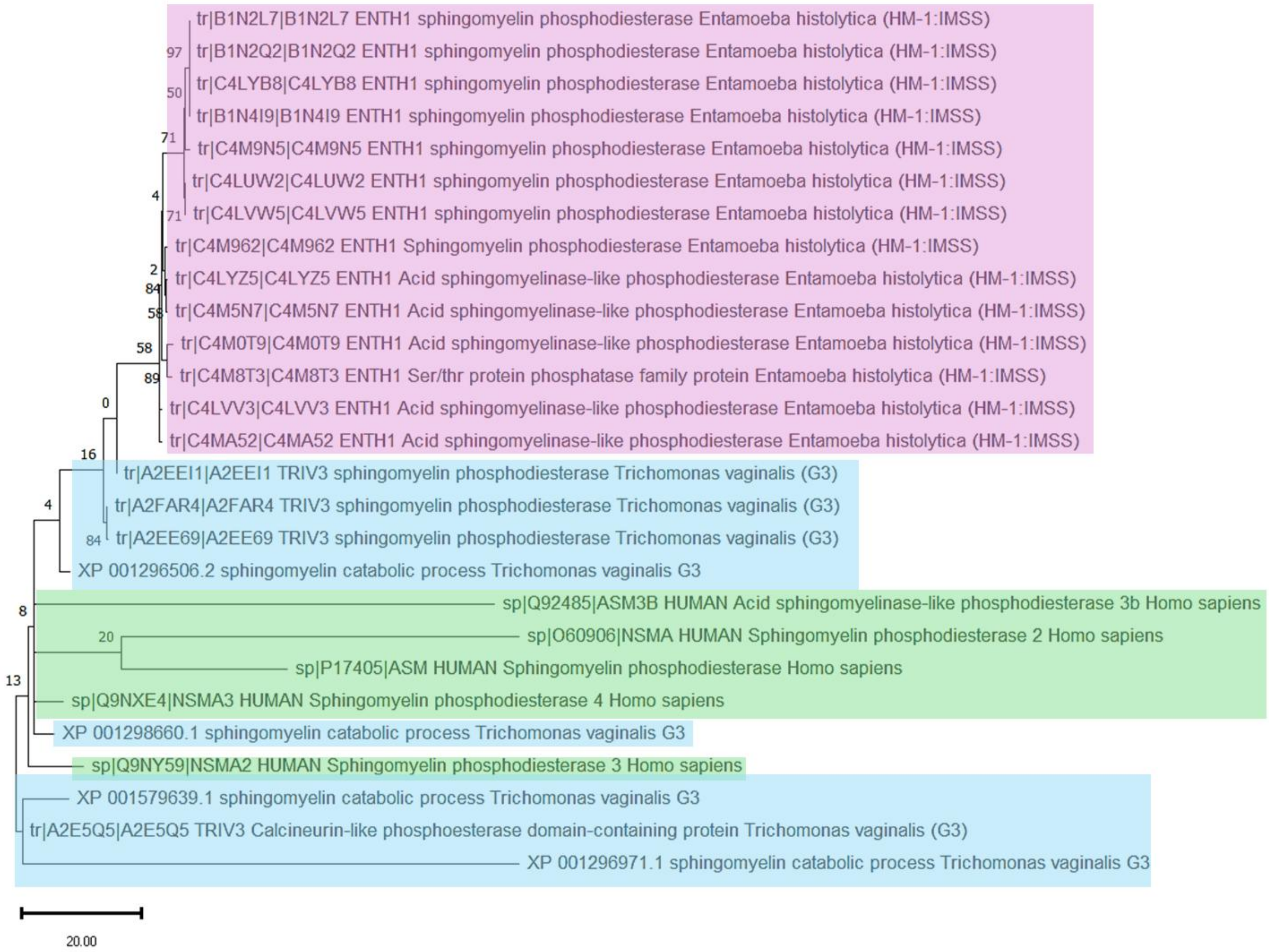

Supplementary Figure S11. Evolutionary relationship between protist enzymes and host enzymes. Human, *E. histolytica* and *T. vaginalis* enzymes were used to assess evolutionary history. History was inferred using the Maximum Likelihood method and JTT matrix-based model [51]. The tree with the highest log likelihood (-27687.76) is shown. The percentage of trees on which the associated taxa clustered together is shown next to the branches. Initial tree(s) for the heuristic search were obtained automatically by applying Neighbor-Join and BioNJ algorithms to a matrix of estimated pairwise distances using the JTT model. Then, the topology with a superior log likelihood value was selected. The tree is drawn to scale, with branch lengths measured in the number of substitutions per site. This analysis involved 27 amino acid sequences. There was a total of 866 positions in the final dataset. The pink box indicates the *E. histolytica* enzymes; in light blue, the *T. vaginalis* enzymes, and in green, the human enzymes.

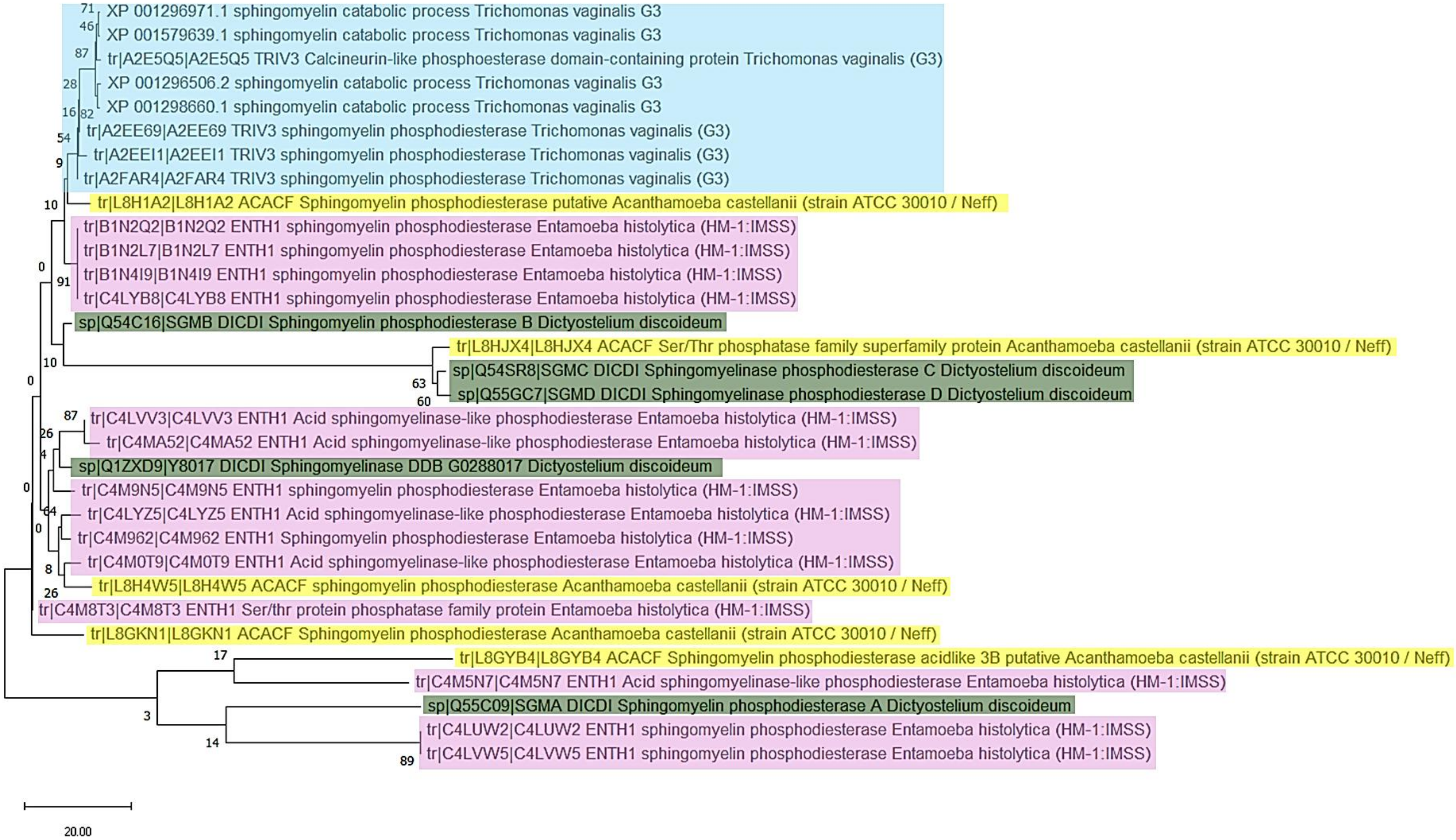

Supplementary Figure S12. All protist evolutionary history also shows no evolutionary relationship with lifestyle. The evolutionary history was inferred using the Maximum Likelihood method and JTT matrix-based model [51]. The tree with the highest log likelihood (-33005.47) is shown. The percentage of trees on which the associated taxa clustered together is shown next to the branches. The heuristic search's initial tree(s) were obtained automatically by applying Neighbor-Join and BioNJ algorithms to a matrix of estimated pairwise distances using the JTT model. Then, the topology with a superior log likelihood value was selected. The tree is drawn to scale, with branch lengths measured in the number of substitutions per site. This analysis involved 32 amino acid sequences. There was a total of 637 positions in the final dataset. In light blue, *T. vaginalis*; yellow, *A. castellanii*; dark green, *D. discoideum*; and in pink, *E. histolytica*

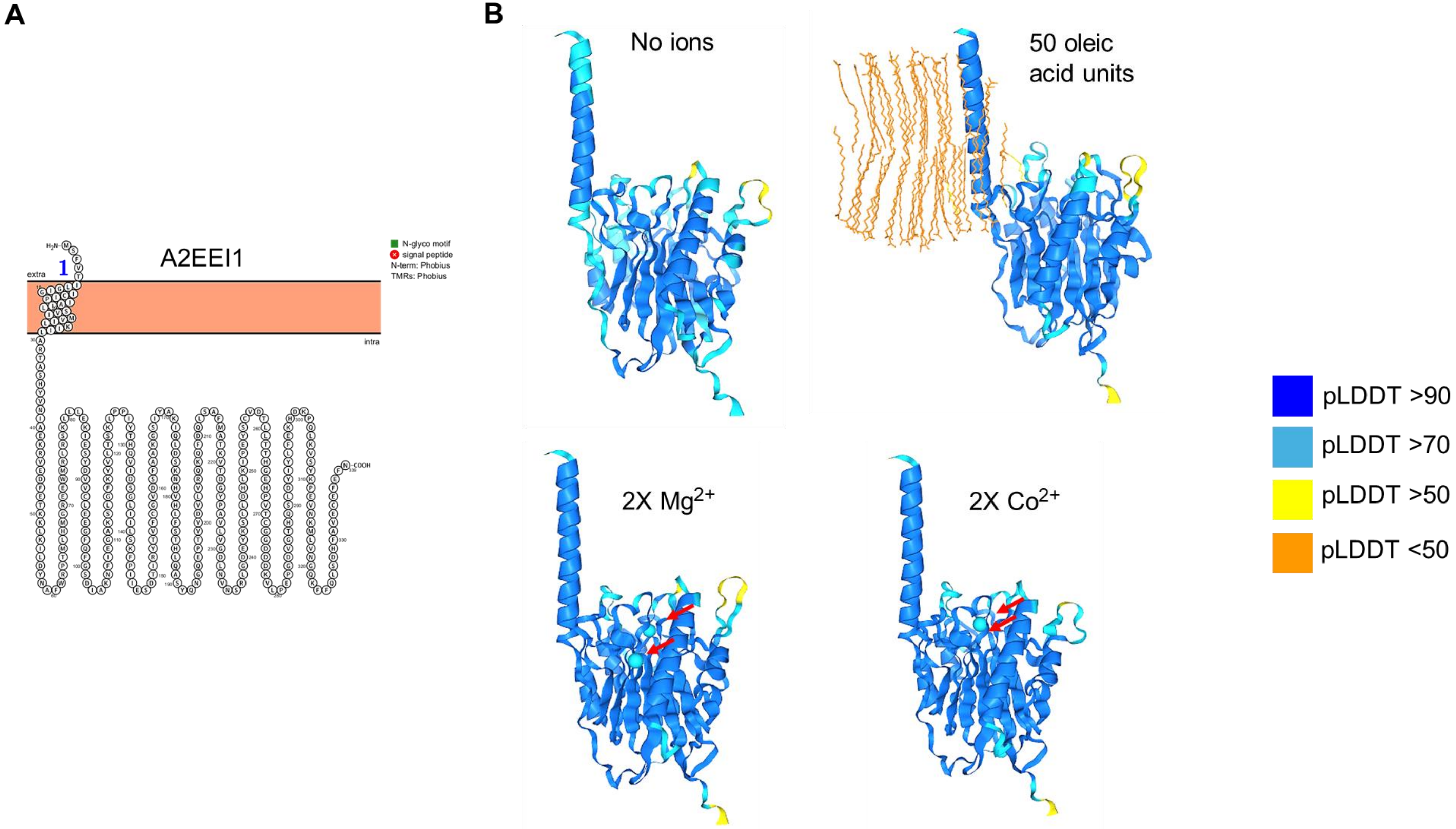

Supplementary Figure S13. *T. vaginalis* SMase accession number A2EEI1 (UniProt) structural features predicted with AlphaFold3. In **Panel A**, Protter's topological prediction is shown. In **Panel B**, the models shown with either metal ions bound (indicated with red arrows) or oleic acid ligands are shown to approximate the predicted membrane-bound domain. The models are shown in the predicted local distance difference test color scheme (pLDDT), and the values are indicated in the figure. Red arrows indicate the position of the metal ions.

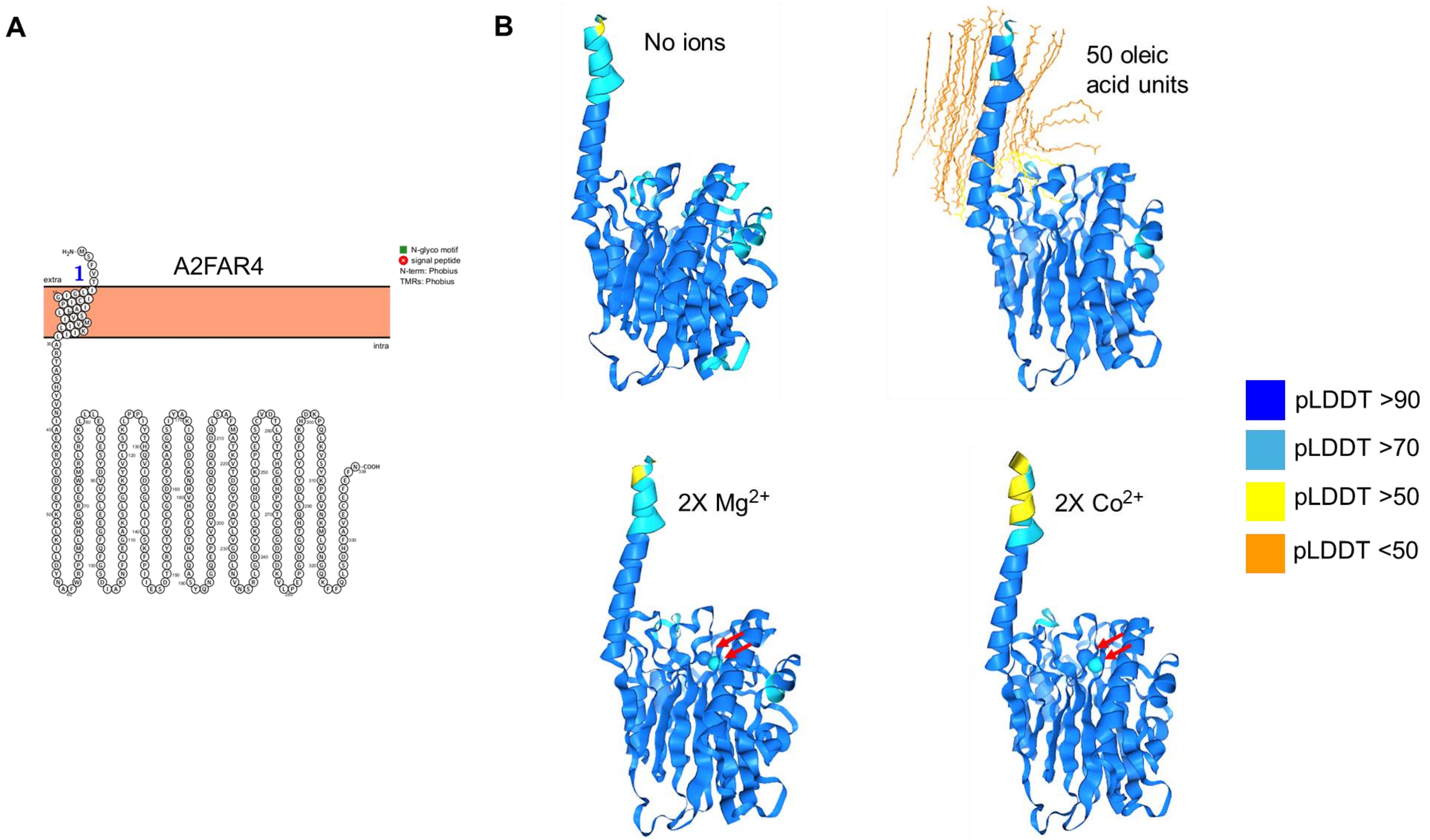

Supplementary Figure S14. *T. vaginalis* SMase accession number A2FAR4 (UniProt) structural features predicted with AlphaFold3. In **Panel A**, Protter's topological prediction is shown. In **Panel B**, the models shown with either metal ions bound (indicated with red arrows) or oleic acid ligands are shown to approximate the predicted membrane-bound domain. The models are shown in the predicted local distance difference test color scheme (pLDDT), and the values are indicated in the figure. Red arrows indicate the position of the metal ions.

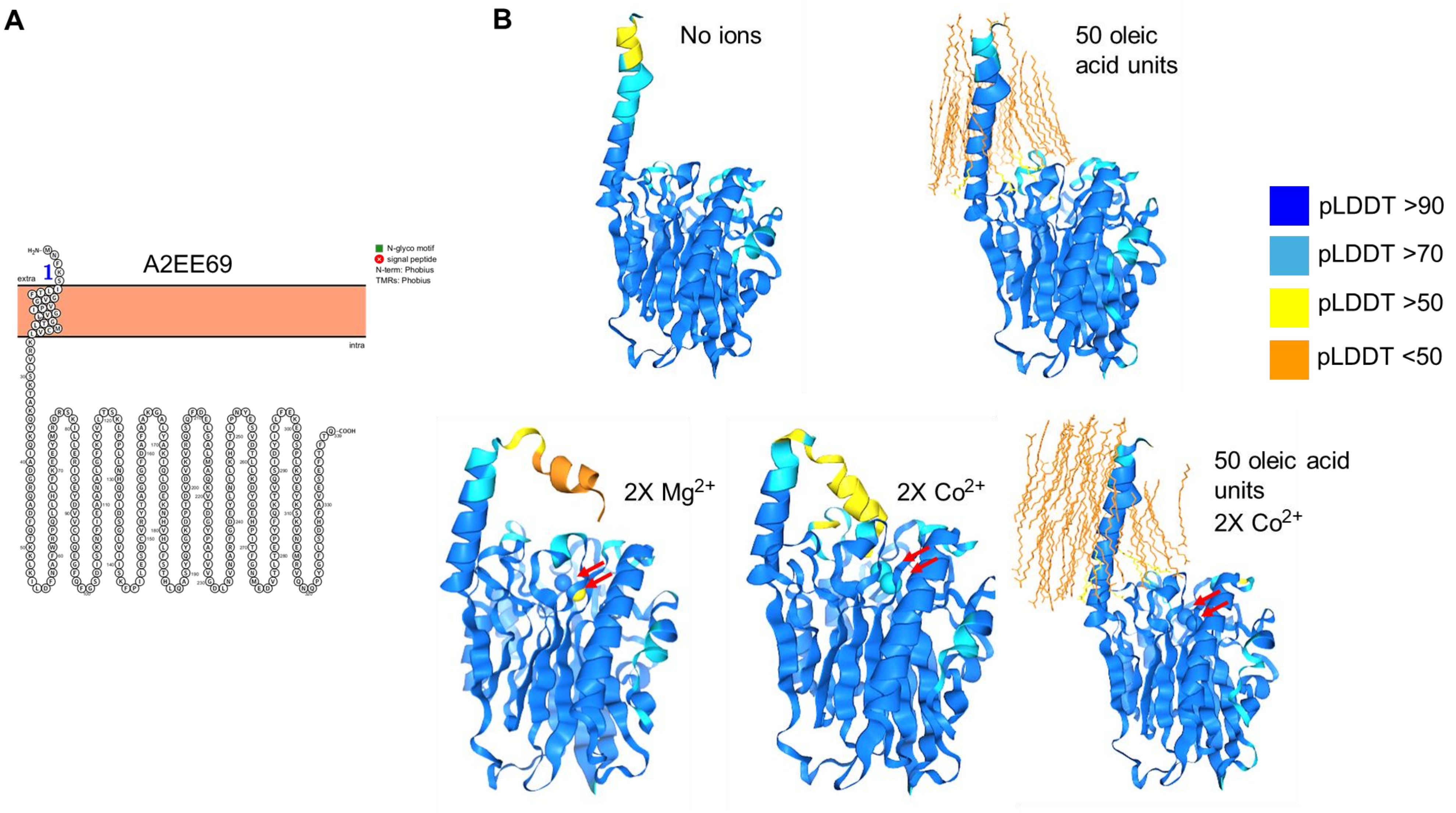

Supplementary Figure S15. *T. vaginalis* SMase accession number A2EE69 (UniProt) structural features predicted with AlphaFold3. In **Panel A**, Protter's topological prediction is shown. In **Panel B**, the models shown with either metal ions bound (indicated with red arrows) or oleic acid ligands are shown to approximate the predicted membrane-bound domain. In these models, the helix showed a different conformation depending on the metal ion bound; thus, the structure was predicted using 50 oleic acid units to evaluate the effect of the ions and the oleic acid. The models are shown in the predicted local distance difference test color scheme (pLDDT), and the values are indicated in the figure. Red arrows indicate the position of the metal ions.

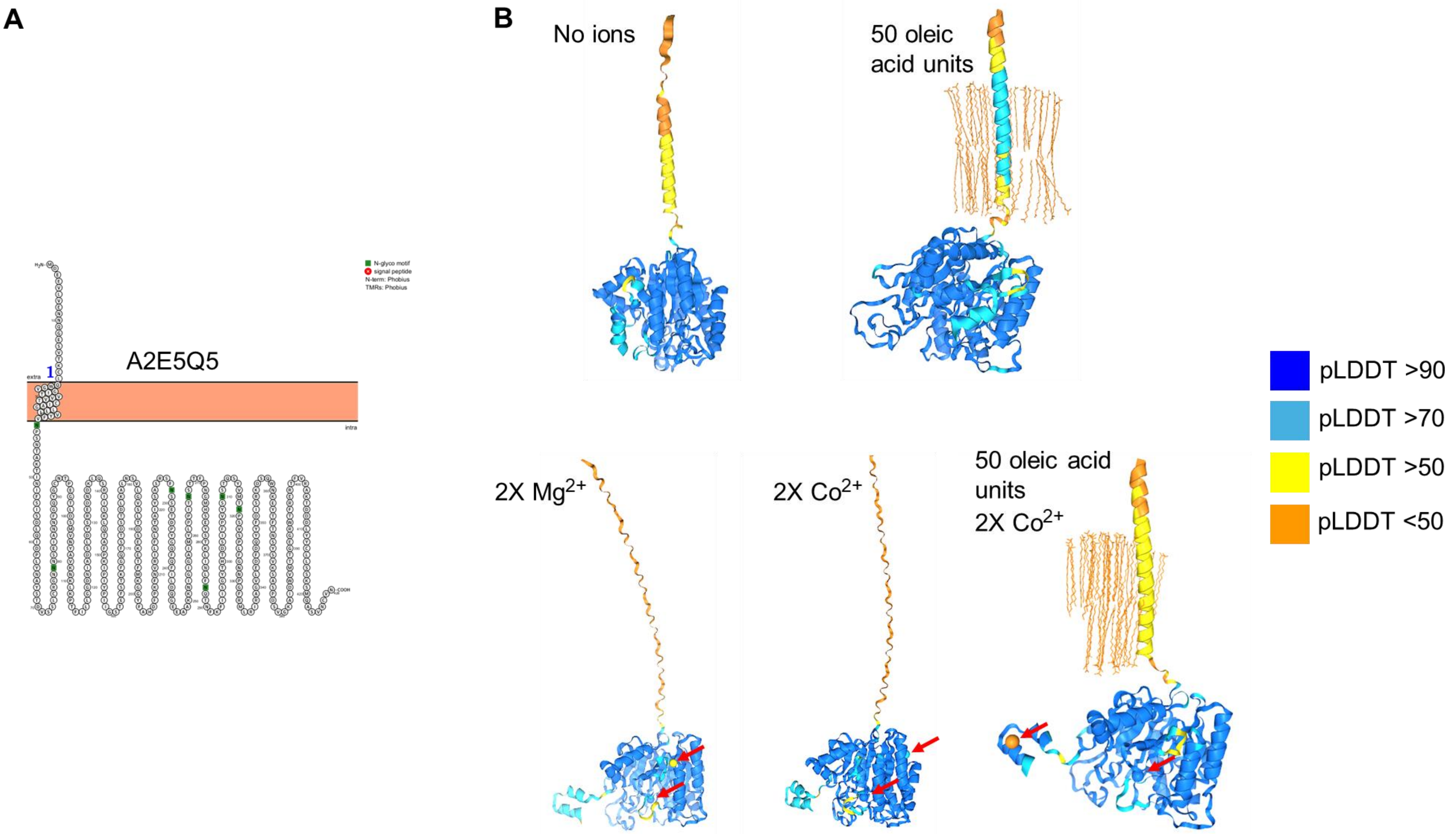

Supplementary Figure S16. *T. vaginalis* SMase accession number A2E5Q5 (UniProt) structural features predicted with AlphaFold3. In **Panel A**, Protter's topological prediction is shown. In **Panel B**, the models shown with either metal ions bound (indicated with red arrows) or oleic acid ligands are shown to approximate the predicted membrane-bound domain. In these models, the helix showed a different conformation depending on the metal ion bound; thus, the structure was predicted using 50 oleic acid units to evaluate the effect of the ions and the oleic acid. The models are shown in the predicted local distance difference test color scheme (pLDDT), and the values are indicated in the figure. Red arrows indicate the position of the metal ions.

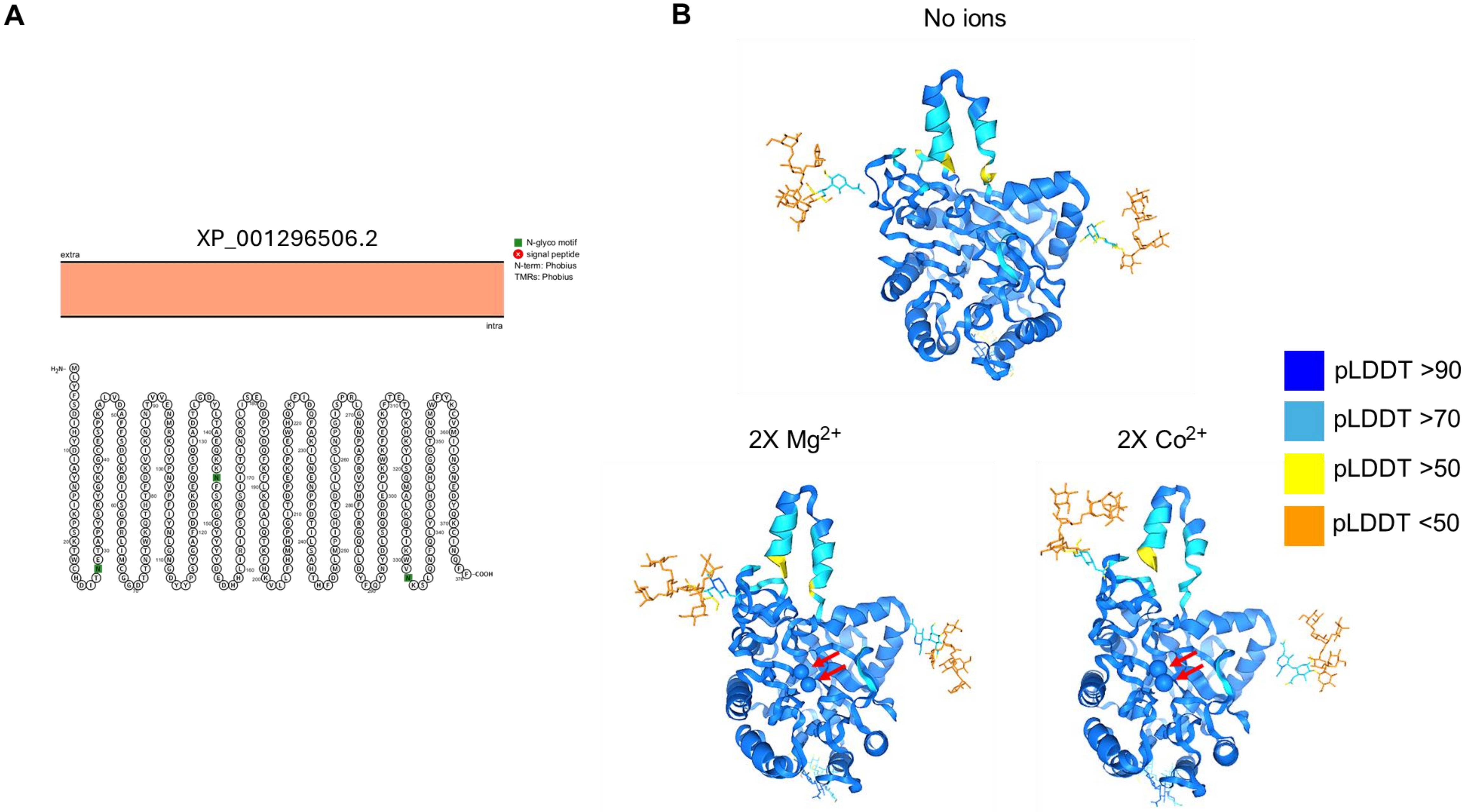

Supplementary Figure S17. *T. vaginalis* SMase accession number XP_001296506.2 (GenBank) structural features predicted with AlphaFold3. In **Panel A**, Protter's topological prediction is shown. In **Panel B**, the models are shown with metal ions bound (indicated with red arrows). The models are shown in the predicted local distance difference test color scheme
[illegible]

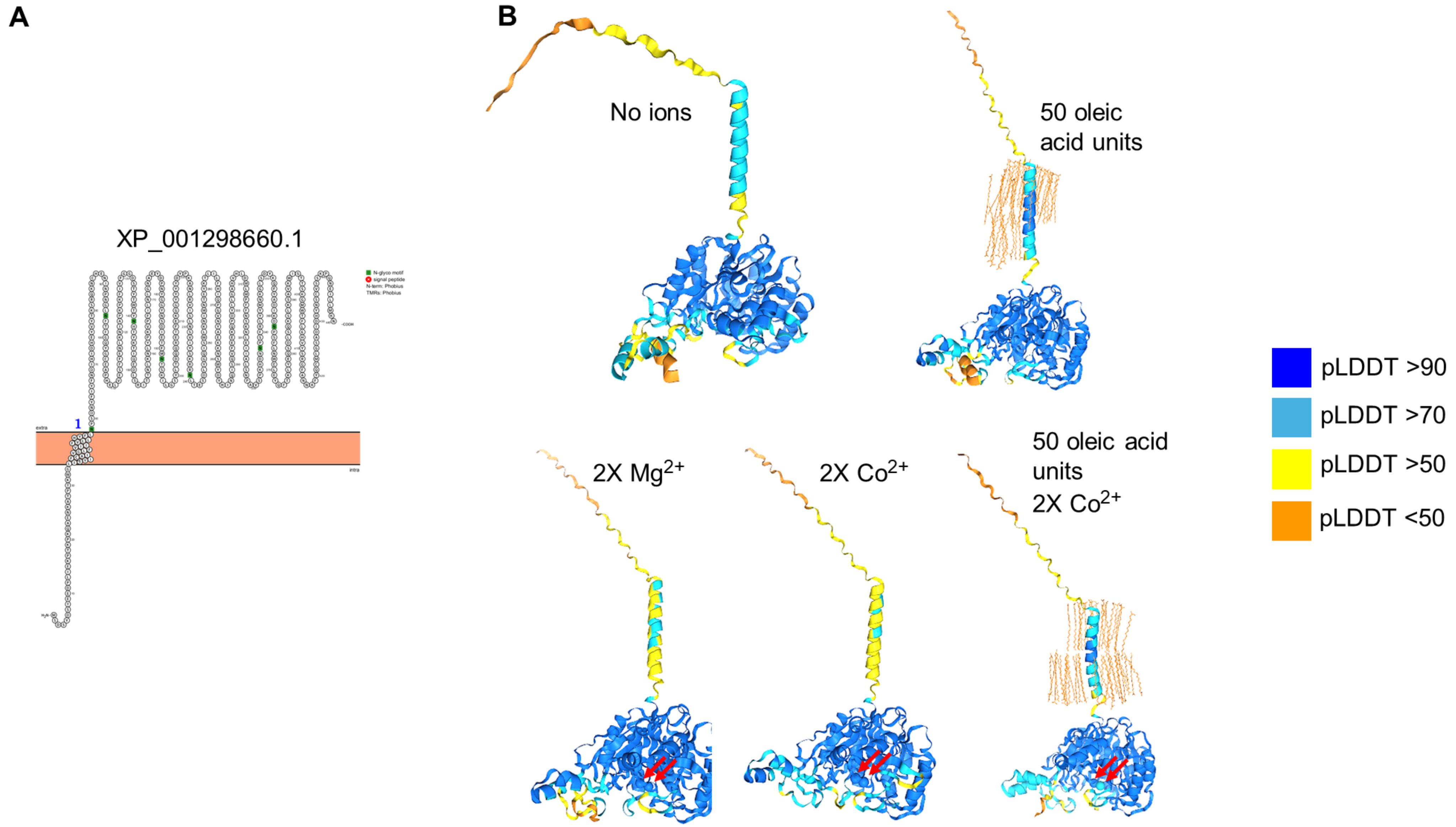

Supplementary Figure S18. *T. vaginalis* SMase accession number XP_001298660.1 (GenBank) structural features predicted with AlphaFold3. In **Panel A**, Protter's topological prediction is shown. In **Panel B**, the models shown with either metal ions bound (indicated with red arrows) or oleic acid ligands are shown to approximate the predicted membrane-bound domain. In these models, the helix showed a different conformation depending on the metal ion bound; thus, the structure was predicted using 50 oleic acid units to evaluate the effect of the ions and the oleic acid. The models are shown in the predicted local distance difference test color scheme (pLDDT), and the values are indicated in the figure. Red arrows indicate the position of the metal ions.

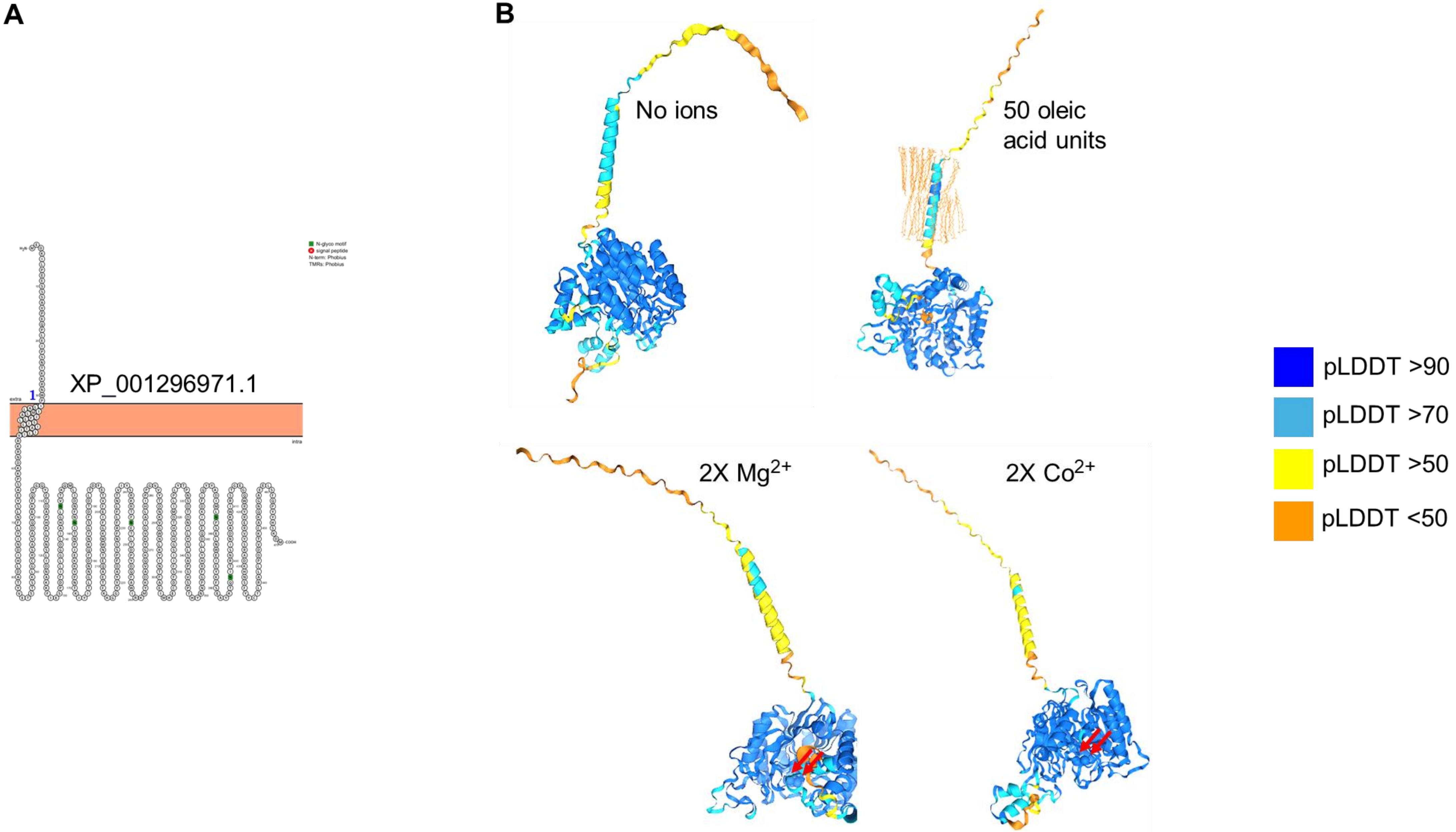

Supplementary Figure S19. *T. vaginalis* SMase accession number XP_001296971.1 (GenBank) structural features predicted with AlphaFold3. In **Panel A**, Protter's topological prediction is shown. In **Panel B**, the models shown with either metal ions bound (indicated with red arrows) or oleic acid ligands are shown to approximate the predicted membrane-bound domain. The models are shown in the predicted local distance difference test color scheme (pLDDT), and the values are indicated in the figure. Red arrows indicate the position of the metal ions.

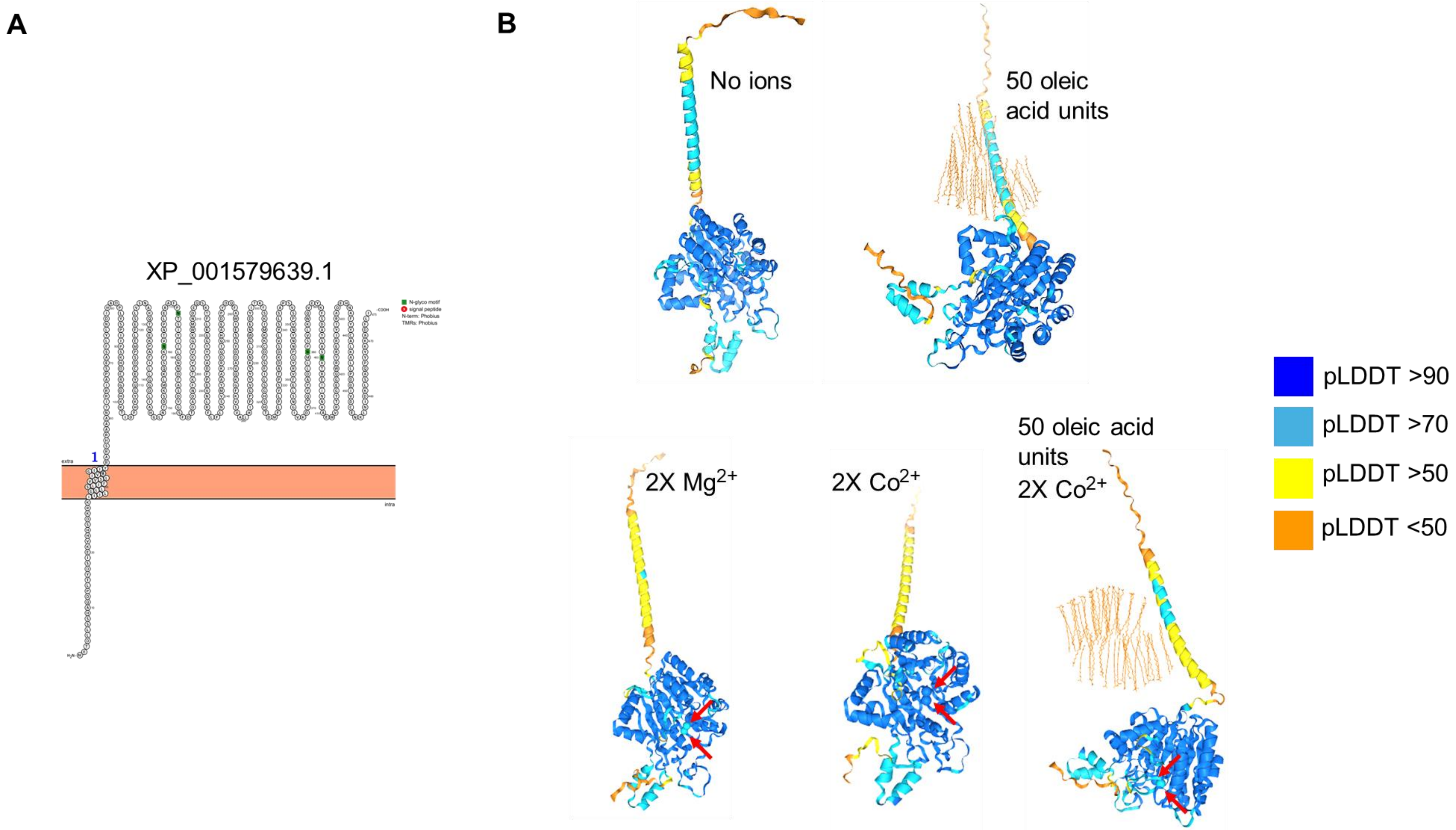

Supplementary Figure S20. *T. vaginalis* SMase accession number XP_001579639.1 (GenBank) structural features predicted with AlphaFold3. In **Panel A**, Protter's topological prediction is shown. In **Panel B**, the models shown with either metal ions bound (indicated with red arrows) or oleic acid ligands are shown to approximate the predicted membrane-bound domain. The models are shown in the predicted local distance difference test color scheme (pLDDT), and the values are indicated in the figure. Red arrows indicate the position of the metal ions.
